# A No-Cost, Bedside, Self-Traction Maneuver for Relief From Chronic Cervical Radiculopathy: A Case Report

**DOI:** 10.7759/cureus.43963

**Published:** 2023-08-23

**Authors:** Rohan G Maharaj, Shastri Motilal, Raveed Khan, Fidel Rampersad

**Affiliations:** 1 Paraclinical Sciences, The University of the West Indies, St. Augustine, TTO; 2 Department of Medicine, The University of the West Indies, St. Augustine, TTO

**Keywords:** case report, traction, pain, neck, spondylosis, cervical

## Abstract

Cervical radiculopathy secondary to spondylosis is common in the elderly. Systematic reviews suggest that no single treatment modality represents the standard of care. A no-cost, bedside, self-traction intervention can be a useful adjunct to current options.

A 60-year-old South Asian male presented with severe cervical radiculopathic pain in April 2019, proven by magnetic resonance imaging to be secondary to spondylosis. Since late 2019, he has been doing a daily self-traction procedure in which he lies prone with the anterior chest and abdomen flat on a bed, shoulders just off the mattress edge, and arms to the side. The position is maintained for 60 seconds, where the weight of the head provides traction. Overhead cervical traction has not been needed for the past three years, and the pain has been bearable. His Neck Disability Index has decreased from 25 to 2. This no-cost, minute-long daily maneuver has provided relief from chronic cervical neuropathic pain due to cervical spondylosis.

## Introduction

Cervical radiculopathy, resulting from spondylosis, is common in older patients. In the USA, the annual incidence of cervical radiculopathy is 107 per 100,000 men and 64 per 100,000 women. It is most common in persons 50-54 years of age, with an incidence of 203 per 100,000 people in this age group [1]. Cervical radiculopathy symptoms include cervical pain which is aggravated by movement. The pain may be referred to the occiput, the shoulder blades, or the upper limbs. Sufferers may also experience retro-orbital or temporal pain (e.g., from spondylosis at C1 to C2). Other symptoms might include cervical stiffness, vague numbness, tingling, weakness in the upper limbs, dizziness, vertigo, and poor balance. Patients can also present with varying levels of myopathy, with weakness of the arm and forearm muscles. Other symptoms such as migraine occur but are uncommon [2]. The most frequent neurologic finding is diminished deep tendon reflexes, particularly of the triceps [3]. As there is no single gold-standard test for cervical radiculopathy, its diagnosis can be made based on clinical findings and radiological investigations [4].

When considering treatment, medical texts recommend a series of interventions, including rest with or without a cervical collar, oral or injected medication, cervical manipulation and traction, exercise, and physiotherapy [5]. Traditional Chinese medicines are also recommended [3]. There are also surgical options, such as anterior cervical discectomy with fusion and cervical disc arthroplasty. These interventions can be cumbersome (e.g., overhead cervical traction), tiresome (e.g., requiring transport to a medical facility), involve other persons (e.g., physiotherapists), or complex and expensive (e.g., cervical surgery). Systematic reviews suggest that no one treatment modality is the standard of care. Mobilization, manipulation, or exercise are reported as being equally effective [2,6-8]. Many sufferers have co-morbidities of diabetes mellitus, cardiovascular disease, and hypertension, making surgical options more complex. In addition, “the outcome of decompressive surgery is often disappointing, especially for myelopathy complicating cervical spondylosis” [2]. Our literature review revealed a case report discussing bedside home traction in a supine position for a 30-year-old patient with non-specific cervical radiculopathy [9]. This underscores the importance of exploring various traction techniques.

Given the current state of ambiguity on the management of cervical radiculopathy secondary to cervical spondylosis, a simple (i.e., no complex contraptions), bedside, convenient (i.e., can be conducted daily at home or when traveling), a no-cost option can be valuable; especially if the option rapidly reduces pain and improves and maintains the patient’s quality of life. Such an alternative would be ideal for a primary care population. It might also be of value in the developing world, where physiotherapy options are limited and/or not covered by insurance, as well as for older patients with medical complications that limit surgical interventions.

This case reports on a simple, no-cost, device-free, minute-long, daily self-traction bedside maneuver for relieving ongoing cervical radiculopathic pain secondary to cervical spondylosis.

## Case presentation

Our patient, whom we call RM here, is a 60-year-old Caribbean male family physician of South Asian descent. He has a past medical history of hyperlipidemia and atherosclerotic heart disease and had two stents inserted in 2013. He was well controlled on anti-lipidemic, anti-platelet, and beta-blocker therapies. He presented on April 16th, 2019, with pain at the root of his left neck of 8/10 severity. There was tingling and numbness over the left deltoid area and the left radial aspect of the arm and forearm extending to the thumb. There was also grade-IV weakness of the left deltoid muscle, as well as fasciculation of the muscles of the left medial forearm. The symptoms were causing decreased sleep and interfered with driving. The pain also affected his work as an academic, which required long hours on the computer and overseas travel; it also affected his social life, as he was unable to attend weekly dance classes with his wife. He had to pay particular attention to his cervical posture, as any cervical flexion or ipsilateral rotation while in the upright position triggered the pain. He calculated that his Neck Disability Index (NDI) then was 25 (50%) representing “severe disability” [10,11].

**Figure 1:**
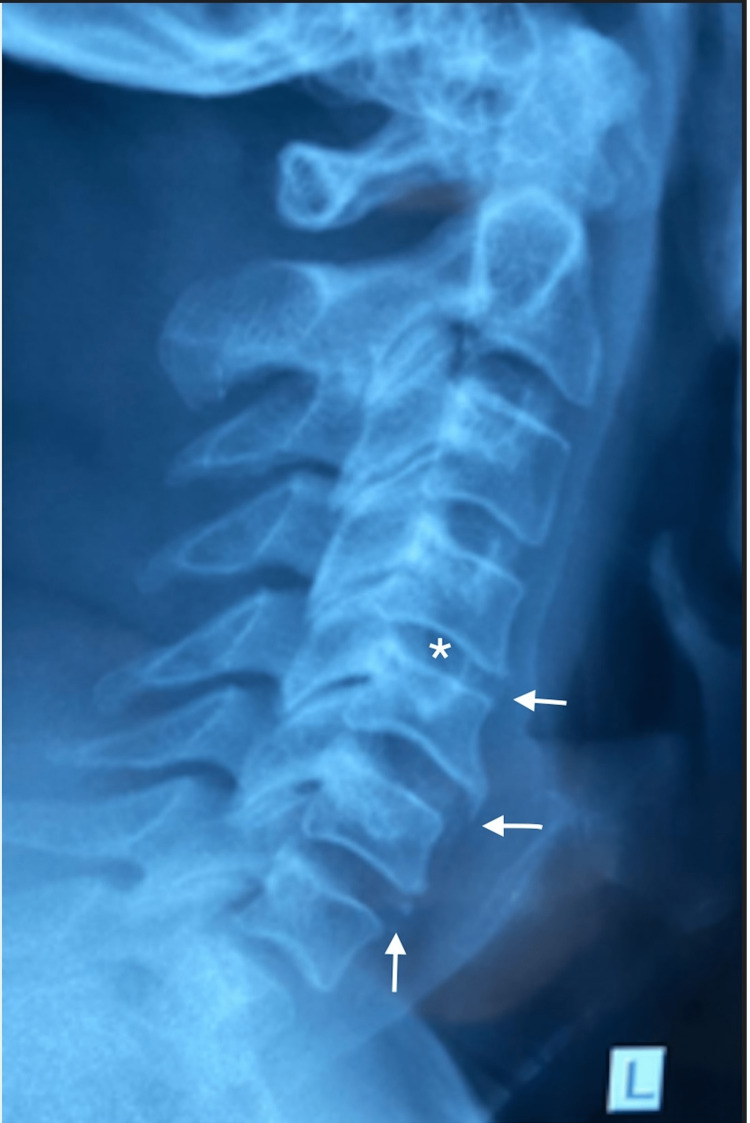
Cervical spine X-ray (lateral view) image Cervical spine X-ray (lateral view) showing loss of the normal cervical lordosis, slight retrolisthesis seen at C4-5 and mild disc height reduction at the C4-5 level (asterisk), anterior osteophytes at the C4-5 and C5-6 levels (horizontal arrow), as well as calcification of the anterior disc annulus at C6-7 (vertical arrow).

An X-ray report from April 17th, 2019, stated that there were “mild spondylotic changes at C5 and C6 and no evidence of neural foramina stenosis” as seen in Figure 1.

**Figure 2:**
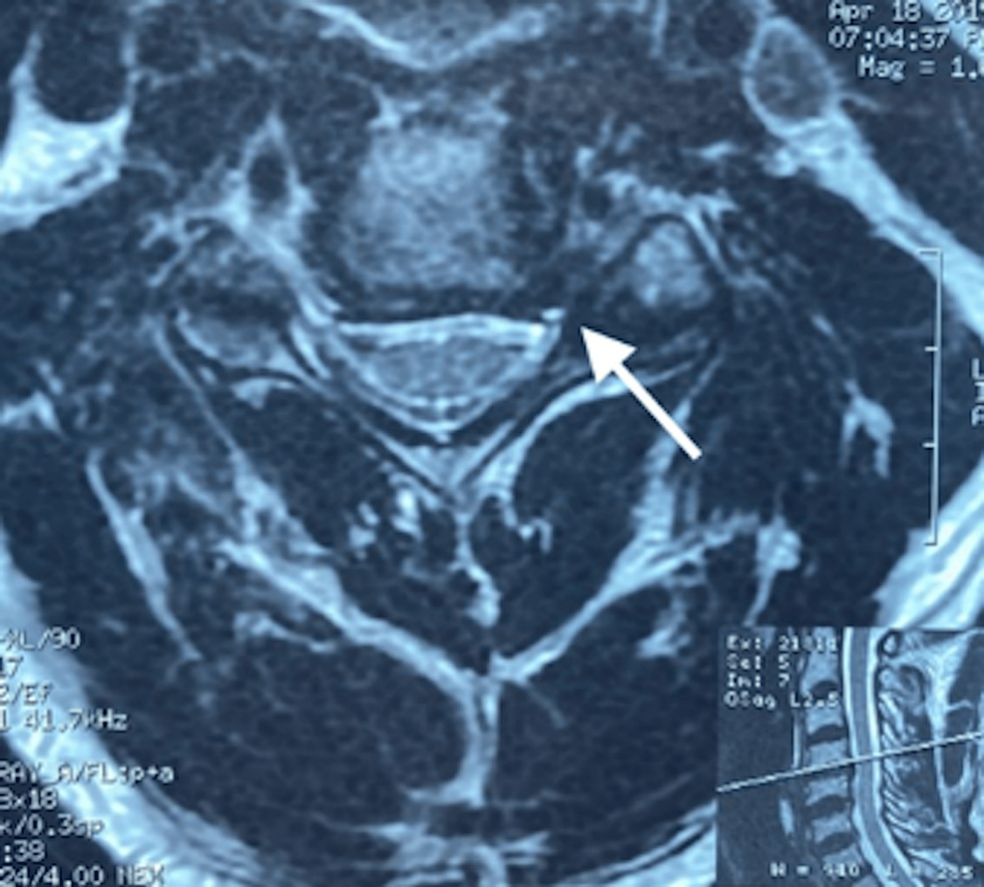
Axial T2 weighted magnetic resonance image at the C4-5 level The image shows left-sided exit foraminal stenosis secondary to uncovertebral osteophytosis, which is a site of exiting left C5 nerve root impingement (arrow). No central spinal canal stenosis is present, nor right-sided exit foraminal stenosis.

**Figure 3:**
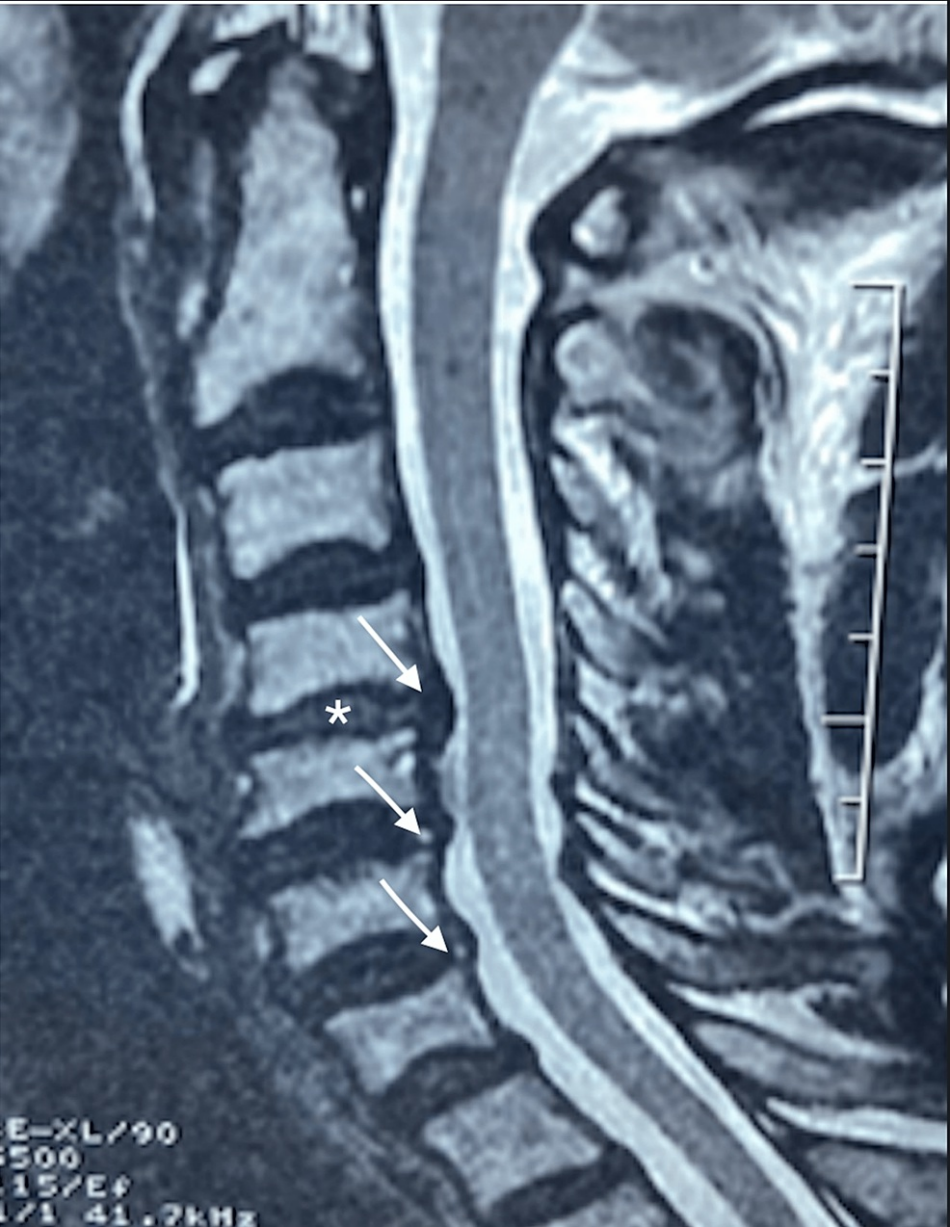
Sagittal T2-weighted magnetic resonance image Sagittal T2-weighted magnetic resonance image revealing loss of the normal cervical lordosis, with endplate changes and osteophytic lipping at the C4-5 and C5-6 levels, loss of disc height at the C4-5 level (asterisk), and posterior osteophyte disc complexes at the C4-5, C5-6, and C6-7 levels (oblique arrows), with no central spinal canal stenosis or cord contact.

A magnetic resonance imaging report on April 18th, 2019, stated that there were “moderate cervical spondylotic changes with mild disc space narrowing at the C4/5 level with mild anterior osteophytic lipping at C4/5 and C5/6 levels. At the C4/5 level, there is an asymmetric narrowing of the epidural fat in the left neural foramen suggesting foraminal stenosis secondary to uncovertebral osteophytes. The severity of the foraminal stenosis suggests irritation of the exiting left C5 root, as seen in Figures 2-3.

RM visited a neurosurgeon on April 26th, 2019, who recommended physiotherapy and overhead cervical traction. There was to be a review with contemplation of surgery if there was no resolution or worsening of the deltoid weakness or physical symptoms. RM underwent eight sessions of overhead cervical traction and physiotherapy sessions between April and August 2019. These interventions gave significant relief, including strengthening the left deltoid. He also started overhead cervical traction at home in June 2019. He continued 30 to 40-minute sessions twice weekly. The pain would return after long workdays, especially when working on the computer. However, the patient found the at-home overhead cervical traction sessions to be cumbersome and often required help from a second person. In addition, there were no opportunities for traction whenever the patient was travelling for work or vacation.

**Figure 4:**
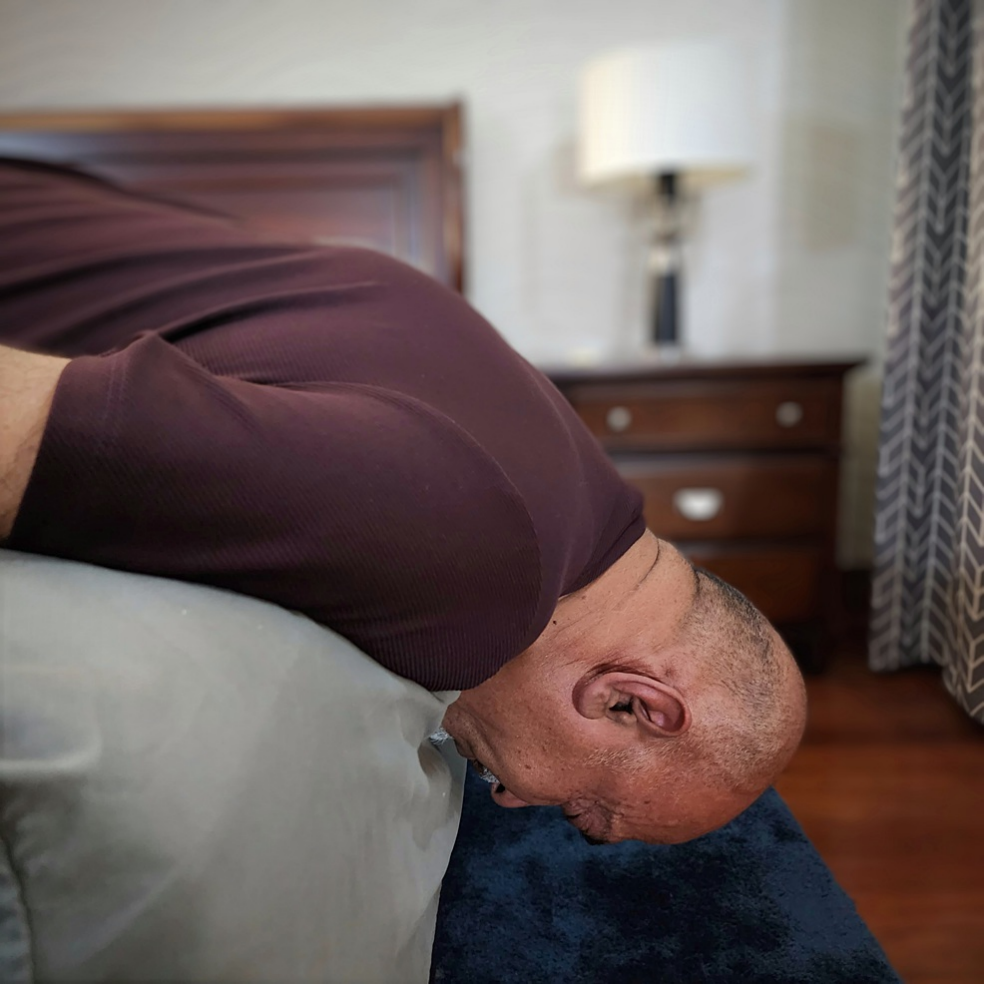
Photo of patient and author (RM) performing bedside cervical traction (used with permission).

After a review of internet resources, RM decided to try a simple no-device technique recommended by two separate YouTube videos [12,13]. The videos recommended this maneuver as part of a series of four or more exercises. However, RM decided to focus on the simplest of these. The sole requirement for this procedure is a supported surface, which is at least 2 feet off the floor. In this self-traction procedure, the patient lies prone, with the anterior chest and abdomen flat on a bed, shoulders off the mattress edge by approximately 2-3 inches (5-7.5 cm), and arms by the side, and the patient suspends their head, neck, and shoulder tops over the mattress edge with their eyes facing towards the bed. The neck is relaxed, and the muscles are loosened. In this maneuver the head’s weight provides traction. Gentle rotation of the neck while in this position helps the muscles relax. The head and neck are maintained in position for 60 seconds, as seen in Figure 4.

The patient experienced three mild side-effects of the maneuver: on one occasion there was worsening of mild vertigo and dizzy spells-a possible side-effect of his beta-blocker treatment or the spondylosis itself. On a second occasion, he experienced gastroesophageal reflux symptoms when the maneuver was tried after a heavy meal. On another occasion after an episode of nasal allergies, RM experienced severe nasal congestion, which required mouth breathing. A comment on YouTube suggested that one participant experienced small, ruptured vessels in their conjunctiva, possibly from hanging their head for too long [14]. At the beginning of attempting the maneuver, it is recommended that the frail, elderly, or persons with poor mobility should be accompanied by a helper. On one occasion, RM reported having a momentary shift in his center of gravity such that he almost fell off the mattress’ edge. One patient with diabetic retinopathy was told that the procedure might have contributed to a retinal bleed (J. Madoo, personal communication, 2023). Patients can expect to experience a soft “pop” in their ears a few seconds into the procedure, which one otolaryngologist suggests may be a referred sound from the loosening of the cervical spine (S. Juman, personal communication, 2022).

## Discussion

RM has been practicing the 60-second maneuver daily for over three years. Since the beginning of this procedure, overhead cervical traction has not been needed. He still pays attention to his cervical posture, and occasionally chin tucks can be helpful [15]. His pain is significantly reduced and only resumes after conducting a long clinical session lasting over 6 hours, or after prolonged computer use. His current NDI is 2 (4%). RM has since recommended this maneuver to his patients with several reporting almost immediate relief of cervical neuropathic pain.

Although this maneuver has provided consistent relief to the patient over the past three years, being a case report, this is very low on the hierarchy of evidence. A clinical trial is planned to determine if this simple maneuver is at least as effective as the current recommended care. For such a trial, the sample size calculations are based on the following parameters: a Type I error of 0.05, a power of 0.8, an allowable difference or true mean difference between the treatment and control groups of zero, an expected population SD of the Neck Disability Index of 20% [11,16], a margin or clinically meaningful difference of 20, and a drop rate of 20%. Given these parameters, the sample size could be as few as 32 participants, with 16 participants in each of the intervention and comparison arms [17].

## Conclusions

Cervical spondylosis and associated neuropathy are common conditions in older patients, causing pain and reduced mobility. In a 60-year-old South Asian male patient, this simple, no-cost, device-free, minute-long daily bedside self-traction has provided ongoing relief from chronic cervical neuropathic pain arising from cervical spondylosis. The sole requirement for this procedure is a supported surface, which is at least 2 feet off the floor. In this self-traction procedure, the patient lies prone, with the anterior chest and abdomen flat on a bed, shoulders off the mattress edge by approximately 2-3 inches (5-7.5 cm), and arms by the side, and the patient suspends their head, neck, and shoulder tops over the mattress edge with their eyes facing towards the bed. The neck is relaxed, and the muscles are loosened. In this maneuver the head’s weight provides traction. While the maneuver has shown effectiveness, it may not be suitable for all patients. Thus, patient selection should be undertaken carefully, and potential problems or contraindications should be thoroughly evaluated.
